# Intrinsic Resistance to 5-Fluorouracil in a Brain Metastatic Variant of Human Breast Cancer Cell Line, MDA-MB-231BR

**DOI:** 10.1371/journal.pone.0164250

**Published:** 2016-10-10

**Authors:** Atsunobu Sagara, Katsuhide Igarashi, Maky Otsuka, Takeshi Karasawa, Noriko Gotoh, Michiko Narita, Naoko Kuzumaki, Minoru Narita, Yoshinori Kato

**Affiliations:** 1 Department of Pharmacology, Hoshi University School of Pharmacy and Pharmaceutical Sciences, 2-4-41 Ebara, Shinagawa-ku, Tokyo, Japan; 2 Life Science Tokyo Advanced Research Center (L-StaR), Hoshi University School of Pharmacy and Pharmaceutical Sciences, 2-4-41 Ebara, Shinagawa-ku, Tokyo, Japan; 3 Division of Cancer Cell Biology, Cancer Research Institute, Kanazawa University, Kakuma-machi, Kanazawa city, Ishikawa, Japan; Universitat Witten/Herdecke, GERMANY

## Abstract

Although drug resistance is often observed in metastatic recurrence of breast cancer, little is known about the intrinsic drug resistance in such metastases. In the present study, we found, for the first time, that MDA-MB-231BR, a brain metastatic variant of a human breast cancer cell line, was refractory to treatment with 5-fluorouracil (5-FU) even without chronic drug exposure, compared to its parent cell line, MDA-MB-231, and a bone metastatic variant, MDA-MB-231SCP2. Both the mRNA and protein levels of COX-2 and BCL2A1 in MDA-MB-231BR were significantly higher than those in MDA-MB-231 or MDA-MB-231SCP2. Neither the COX-2 inhibitor celecoxib nor the NF-κB inhibitor BAY11-7082 could sensitize MDA-MB-231BR to 5-FU, indicating that COX-2 plays little, if any, role in the resistance of MDA-MB-231BR to 5-FU. Although BCL2-family inhibitor ABT-263 failed to sensitize MDA-MB-231BR to 5-FU at a dose at which ABT-263 is considered to bind to BCL2, BCL2-xL, and BCL2-w, but not to BCL2A1, ABT-263 did sensitize MDA-MB-231BR to 5-FU to a level comparable to that in MDA-MB-231 at a dose of 5 μM, at which ABT-263 may disrupt intracellular BCL2A1 protein interactions. More importantly, *BCL2A1* siRNA sensitized MDA-MB-231BR to 5-FU, whereas the overexpression of *BCL2A1* conferred 5-FU-resistance on MDA-MB-231. These results indicate that BCL2A1 is a key contributor to the intrinsic 5-FU-resistance in MDA-MB-231BR. It is interesting to note that the drug sensitivity of MDA-MB-231BR was distinct from that of MDA-MB-231SCP2 even though they have the same origin (MDA-MB-231). Further investigations pertinent to the present findings may provide valuable insight into the breast cancer brain metastasis.

## Introduction

Breast cancer is the most common malignancy among women worldwide. Despite recent advances in targeted cancer therapies, breast cancer is still the second most frequent cause of death in women [1]. Patients with triple-negative breast cancers (TNBC), characterized by the absence of estrogen receptor, a progesterone receptor, and a human epidermal growth factor receptor type 2 (HER2) expression, make up approximately 12 to 17% of the total number of breast cancer patients and have a relatively poor prognosis. This hallmark makes TNBC difficult to treat by hormonal or anti-HER2 therapy, and only classical cytotoxic agents, such as 5-fluorouracil (5-FU), offer a viable option to those who develop distant metastasis. However, in addition to their toxic side effects, classical cytotoxic agents often pose serious problems, including multi-drug resistance and/or metastatic recurrence. These events are considered to be associated with the overexpression of drug efflux transporters/certain enzymes, or the epithelial-mesenchymal transition (EMT) [2]. Drug resistance following chronic treatment with anticancer agents has been reported to trigger cancer metastasis [3, 4], although metastasis can occur even prior to chemotherapy. In addition, breast cancer cells found at metastatic sites may or may not be refractory to chemotherapy, regardless of prior exposure to anticancer agents; i.e., metastatic breast cancer cells may develop drug resistance spontaneously. Despite extensive research on drug resistance and breast cancer metastasis, little is known about their relationship and particularly that between intrinsic drug resistance and breast cancer metastasis. Considering that both drug resistance and metastasis are factors for a poor prognosis in breast cancer patients, it would be important to investigate the relationship between intrinsic drug resistance and breast cancer metastasis.

MDA-MB-231 is a highly invasive human breast cancer cell line that has been used as a human TNBC cell line for more than three decades. Its metastatic variants MDA-MB-231BR and MDA-MB-231SCP2 are increasingly gaining attention as a brain metastatic phenotype and a bone metastatic phenotype, respectively. The vast majority of preclinical research that has compared these cell lines has focused on factors related to metastasis and, to the best of our knowledge, there have been no reports on drug resistance in these cell lines. In the present study, we discovered that MDA-MB-231BR was refractory to 5-FU in comparison to both its parent cell line, MDA-MB-231, and MDA-MB-231SCP2. We also found that the intrinsic overexpression of BCL2A1 contributed to 5-FU-resistance in MDA-MB-231BR.

## Materials and Methods

### Cell Culture

The human breast carcinoma cell lines MDA-MB-231 (ECACC, Salisbury, UK), a brain metastatic variant MDA-MB-231BR, a bone metastatic variant MDA-MB-231SCP2, MCF-7, and T-47D (ECACC, Salisbury, UK) were cultured in RPMI-1640 (ThermoFisher Scientific Inc., Waltham, MA) with 10% fetal bovine serum. MDA-MB-231BR and MDA-MB-231SCP2/TGL (MDA-MB-231SCP2) were kind gifts from Dr. Patricia Steeg and Dr. Joan Massagué, respectively. The human mammary epithelial cell line MCF-10A was purchased from ATCC (Manassas, VA), and cultured in MEBM Basal Medium (Lonza, Basel, Switzerland) with 100 ng/mL cholera toxin and the provided supplements. The human glioblastoma cell line U87MG was a kind gift from Dr. Dmitri Artemov (the Johns Hopkins University School of Medicine), and cultured in MEM-Alpha (ThermoFisher) with 10% fetal bovine serum and glucose at a final concentration of 4.5 g/L. Contamination with *Mycoplasma* or fungi was routinely checked and the cells were treated or discarded when necessary; only uncontaminated cells were used. All cells were maintained under a humidified atmosphere of 5% CO_2_ at 37°C.

### Reagents

5-Fluorouracil (5-FU) and BAY11-7082 were purchased from Wako Pure Chemical Industries, Ltd. (Osaka, Japan). Celecoxib was obtained from Tokyo Chemical Industry Co., Ltd. (Tokyo, Japan). ABT-263 was obtained from Selleck Chemicals (Houston, TX).

### Cytotoxicity assay

A cytotoxicity study was performed using a Cell Counting Kit-8 (CCK-8; Dojindo Laboratories, Kumamoto, Japan) in accordance with the manufacturer's instructions. Each cell line (2×10^3^ cells/well for T-47D; 5×10^3^ cells/well for the other cell lines) was treated with various concentrations of 5-FU or doxorubicin for 48 hrs, and cell viability was measured at 450 nm using a microplate reader (BioRad, Hercules, CA) after a 4-hour incubation with CCK-8 reagent. IC_50_ values were calculated on GraphPad Prism software (San Diego, CA). For the inhibition experiment, inhibitors (50 μM celecoxib, 1 μM BAY11-7082, or 0.3 and 5 μM ABT-263) were added to the cells along with 5-FU. Since higher concentrations of BAY11-7082 (3 and 10 μM) had cytotoxicity by itself, which would overestimate the combination effect of BAY11-7082 and 5-FU, we used BAY11-7082 at a concentration of 1 μM only. The cytotoxicity experiments were performed at least in quadruplicate.

### Flow cytometry

Since drug resistant human breast cancer cells are thought to have a large population of cancer stem cells [5], CD44 and CD24 expression in MDA-MB-231 and its metastatic variants was evaluated with flow cytometry using a double-staining technique. Briefly, each cell line was incubated with PerCP-Cy5.5-conjugated anti-human CD44 antibody (BD Biosciences, San Jose, CA) for 30 min, followed by PE-conjugated anti-human CD24 antibody (Abcam, Cambridge, MA) for 30 min. Flow cytometry was performed using a BD FACSVerse^™^ flow cytometer (BD Biosciences).

### RNA sequencing

Total RNA was isolated from each cell line using a mirVana^™^ Isolation Kit (ThermoFisher Scientific Inc.), according to the manufacturer's instructions. Libraries for RNA-seq were constructed using SMARTer Stranded Total RNA-Seq Kit-Pico Input Mammalian (Takara Bio, Tokyo, Japan) according to the manufacturer's instructions. Ten nanograms of total RNA were transcribed into first-strand cDNA with SMARTScribe Reverse Transcriptase (RT) and N6 Primer (random primer). RT adds additional nucleotides to the 3’ end of cDNA, while the Template Switching Oligo (TSO) hybridizes to the added nucleotides, and RT continues replicating to the end of TSO. The resulting cDNA consisted of 5’ random primer sequences and 3’ specific sequences for the annealing of PCR primer used in the next step. Several cycles of PCR amplification were carried out to add Illumina adaptors and barcodes. In the presence of R-probes that hybridize to ribosomal RNAs (rRNAs), double-stranded DNAs derived from rRNAs were cleaved with ZapR. The remaining double-stranded DNAs were amplified by PCR (14 cycles) with SeqAmp DNA Polymerase to obtain RNA-seq libraries sufficient for sequencing. Libraries were purified with AMPuteXP beads (Beckman Coulter, Inc., Brea, CA), validated using a Tapestation 2200 system (Agilent Technologies, Inc., Santa Clara, CA), and quantitated with a QuantiFluor dsDNA System (Promega Corp., Madison, WI). All libraries were sequenced on Miseq (Illumina, Inc., San Diego, CA) with paired-end read (75 bp). The 75-bp-long paired-end sequence reads were mapped to a human genome reference sequence (hg19) using CLC Genomics Workbench software (CLC bio, Aarhus, Denmark), and the mapped data were exported as BAM files and imported to Strand NGS analysis software (Agilent Technologies). For downstream gene expression analysis, the expression level of genes was quantitated by counting the number of reads mapped to the genes. The raw counts were normalized with DESeq, and differentially expressed genes were manually selected.

### Quantitative reverse transcription PCR (qRT-PCR)

Total RNA was isolated from each cell line using RNeasy (QIAGEN, Hilden, Germany), according to the manufacturer's instructions. Reverse-transcription was performed with the PrimeScript RT Master Mix (Takara Bio Inc., Shiga, Japan). The PCR primer sets used are shown in S1 Table. qPCR was performed using SYBR Premix Ex Taq II (Takara Bio) and an Applied Biosystems^®^ StepOnePlus^™^ Real-Time PCR System (ThermoFisher). The thermal cycle profile was: 1) activating at 95°C for 30 sec; 2) denaturing at 95°C for 5 sec; and 3) annealing and extension at 60°C for 30 sec. PCR amplification was performed for 40 cycles. We used RPS18, GAPDH, HPRT1, and RPLP2 mRNAs as reference genes, and data were expressed as the expression relative to RPS18 mRNA as a housekeeping gene using the 2^-deltadeltaCT^ method because *Ct* values of RPS18 mRNA were most constant among cell lines used.

### Immunoblotting

Cells were solubilized with a cell lysis buffer (Cell Signaling Technology, Danvers, MA), 1 mM phenylmethylsulfonyl fluoride (PMSF; Cell Signaling Technology), and a protease/phosphatase inhibitor cocktail (Cell Signaling Technology) for 30 min at 4°C. Cell lysates were centrifuged at 14,000 ×*g* for 20 min at 4°C, and the supernatant was used for Western blotting. After quantification of protein concentration each sample, the samples (50 μg of total protein per lane) were run on 12% SDS-PAGE gels (BioRad, Hercules, CA) and immunoprobed with COX-2 and BCL2A1 protein. Results were visualized by SuperSignal^™^ West Femto chemiluminescent substrate (ThermoFisher Scientific, Waltham, MA). Rabbit monoclonal antibodies for COX-2 (Cell Signaling Technology) and BCL2A1 (Abcam, Cambridge, UK) were used at 1:1,000 and 1:500 dilutions, and anti-rabbit IgG secondary antibody was used at 1:20,000 dilution (Cell Signaling Technology). Antibody against β-actin (Cell Signaling Technology) at a dilution of 1:1,000 was used as a loading control. Signals were detected with an Amersham Imager 600 (GE Healthcare).

### Enzyme-linked immunosorbent assay (ELISA) for BCL2A1 protein

Cell pellets were collected, lysed with 1X RIPA buffer, and cell lysates used for ELISA to determine BCL2A1 proteins in MDA-MB-231, MDA-MB-231SCP2, and MDA-MB-231BR, which was carried out using a human BCL2A1 ELISA kit (MyBioSource, Inc., San Diego, CA) according to the manufacturer's instructions. At least five independent samples were collected and used for the assay.

### siRNA delivery

SMARTpool: Accell Human *BCL2A1* siRNA (GE Healthcare Dharmacon Inc., Lafayette, CO) or control siRNA were transfected into MDA-MB-231BR at a final concentration of 1 μM with Accell siRNA delivery media with 2.5% FBS. Complexes of siRNA were added to MDA-MB-231BR cells cultured in 24-well and 96-well plates to determine BCL2A1 mRNA/protein expressions and the cytotoxicity of 5-FU, respectively, after transfection with *BCL2A1* siRNA. The knockdown efficiency of BCL2A1 mRNA and protein and cytotoxicity were assessed by a quantitative RT-PCR, Western blot, and a CCK-8 assay, respectively, all of which were carried out 72 hr after transfection. Triplicate independent samples were collected and used for each experiment.

### Transfection of BCL2A1 vector into MDA-MB-231 and T-47D

pCMV-Myc-DDK-Entry vector or pCMV-Myc-DDK-*BCL2A1* vector (Origene Technologies, Inc., Rockville, MD) was transfected into MDA-MB-231 and T-47D using Lipofectamine^®^ LTX, PLUS^™^ reagent, and Opti-MEM reduced serum media, according to the manufacturer's protocol (ThermoFisher Scientific Inc.). Complexes of a plasmid vector were added to MDA-MB-231 and T-47D cells cultured in 24-well plates and 96-well plates to determine BCL2A1 mRNA/protein expressions and the cytotoxicity of 5-FU, respectively, after transfection with the plasmid vector. BCL2A1 mRNA and protein expressions and the cytotoxicity were assessed by a quantitative RT-PCR, Western blot, and a CCK-8 assay, respectively. At least triplicate independent samples were collected and used for each experiment.

### Statistical analysis

The statistical significance of differences was determined by one-way analysis of variance (ANOVA) with the Bonferroni post hoc analysis or a one-tailed Student's *t*-test using StatPlus^®^:mac (AnalystSoft Inc., Alexandria, VA, U.S.A.) software. A value of *P*<0.05 was considered significant.

## Results

### MDA-MB-231BR exhibits intrinsic resistance to 5-FU compared to MDA-MB-231 and MDA-MB-231SCP2

**Fig 1:**
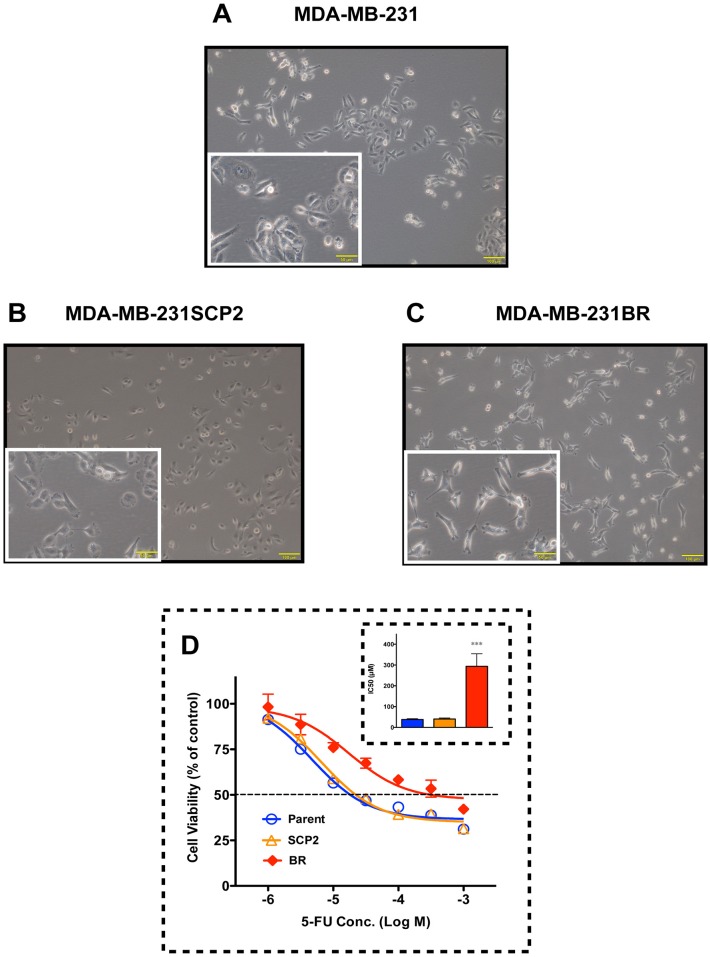
Morphological differences among MDA-MB-231 cell lines, and their cytotoxic response to 5-FU. (A-C) Photomicrographs of (A) MDA-MB-231, (B) MDA-MB-231SCP2, and (C) MDA-MB-231BR. Scale bar, 100 μm. Scale bars in the insets indicate 50 μm. (D) Cytotoxicity of 5-FU toward MDA-MB-231 (Parent), MDA-MB-231SCP2 (SCP2), and MDA-MB-231BR (BR). The inset shows the IC_50_ of 5-FU against each cell line. Data represent the mean with SEM of at least six independent samples (***p<0.001 *vs*. Parent and SCP2).

Although MDA-MB-231BR and MDA-MB-231SCP2 have the same origin (MDA-MB-231), three cell lines were morphologically different (Fig 1A–1C). Compared to MDA-MB-231, MDA-MB-231BR was shaped like a nudibranch while MDA-MB-231SCP2 had a rounded shape. This observation prompted us to explore the drug sensitivity of the cell lines to classical cytotoxic anticancer agents, 5-FU and doxorubicin. Interestingly, intrinsic resistance to 5-FU was observed in MDA-MB-231BR, but not in MDA-MB-231SCP2 (Fig 1D). The IC_50_ values of 5-FU against MDA-MB-231, MDA-MB-231SCP2, and MDA-MB-231BR were 38.2 μM, 40.7 μM and 293.8 μM, respectively. All three cell lines were equally sensitive to doxorubicin (S1 Fig).

### Gene and protein expressions of COX-2 and BCL2A1 in MDA-MB-231BR were significantly higher than those in MDA-MB-231 and MDA-MB-231SCP2

**Fig 2:**
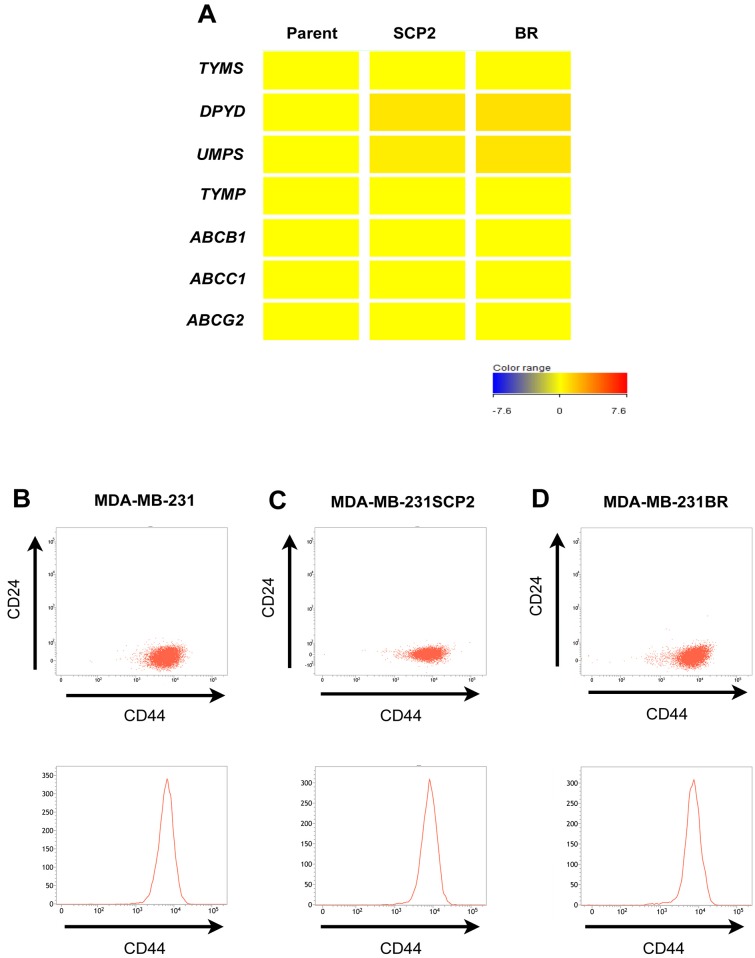
Gene expressions and breast cancer stemness in MDA-MB-231 and its metastatic variants. (A) Gene expression levels in MDA-MB-231 (Parent), MDA-MB-231SCP2 (SCP2), and MDA-MB-231BR (BR). Candidate genes for drug resistance were screened by RNA sequencing. The intensity of gene expression in the heat map is shown relative to that in the Parent. (B-D) Flow cytometry analysis of MDA-MB-231 and its metastatic variants. (B) MDA-MB-231. (C) MDA-MB-231SCP2. (D) MDA-MB-231BR. Both scatter plots and histograms demonstrate that the CD44^+^/CD24^-^ populations of the two metastatic variants were not different from those of their parent cell line.

**Fig 3:**
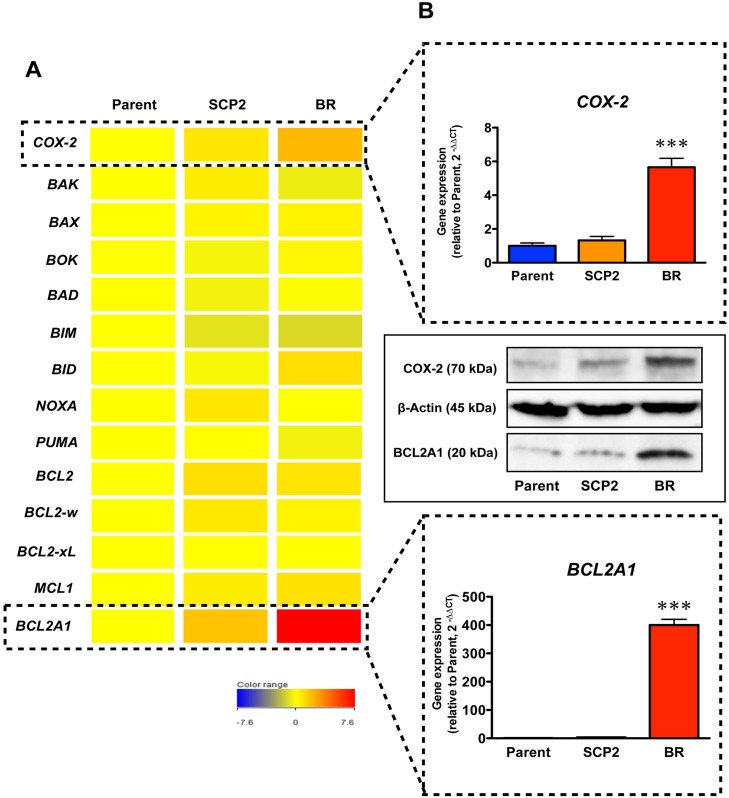
Elevated gene and protein expressions of COX-2 and BCL2A1 in MDA-MB-231BR. (A) Gene expression levels in MDA-MB-231 (Parent), MDA-MB-231SCP2 (SCP2), and MDA-MB-231BR (BR). Candidate genes for drug resistance were screened by RNA sequencing. The intensity of gene expression in the heat map is shown relative to that in the Parent. (B) COX-2 and BCL2A1 mRNA and protein expression levels in Parent, SCP2, and BR. mRNA expression levels in the SCP2 and BR are shown relative to those in the Parent. Data represent the mean with SEM of at least three independent samples (***P<0.001 *vs*. Parent and SCP2) for mRNA expressions.

Next, to identify candidate genes that play an important role in drug resistance in MDA-MB-231BR, we performed RNA sequencing using these cell lines. In contrast to our expectations, there was little difference in the expression of genes related to 5-FU metabolism (Fig 2A). We then checked the expression of drug efflux transporters and breast cancer stem cell markers CD44^+^/CD24^-^, both of which are commonly considered to be key players in drug resistance; there were no differences in the expression of drug efflux transporters (Fig 2A) or CD44^+^/CD24^-^ populations (Fig 2B–2D). Among genes related to 5-FU-resistance and cell death, the expression levels of *COX-2* (*PTGS2*) and the anti-apoptotic gene *BCL2A1* were significantly elevated in MDA-MB-231BR relative to MDA-MB-231 (Fig 3). Similarly, these proteins expressed in MDA-MB-231BR were greater than that in the other cell lines (Fig 3B and S2 Fig). Gene expression levels of other pro- or anti-apoptic genes in the BCL2 family in MDA-MB-231BR relative to those in MDA-MB-231 were marginal.

### BCL2A1 was a key contributor to 5-FU-resistance in MDA-MB-231BR

**Fig 4:**
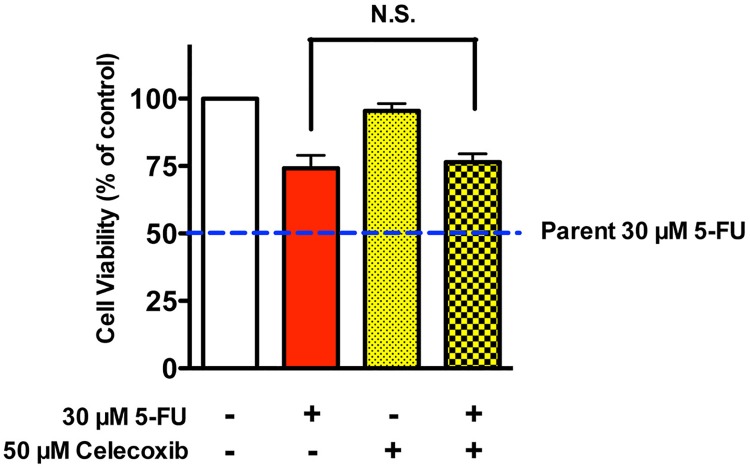
Effect of celecoxib on the cytotoxicity of 5-FU against MDA-MB-231BR. Cytotoxicity of 5-FU to MDA-MB-231BR in the presence of 50 μM celecoxib. Blue broken lines parallel to x-axis represent the cell viability of MDA-MB-231 (Parent) treated with 30 μM 5-FU. Data represent the mean with SEM of four independent samples.

**Fig 5:**
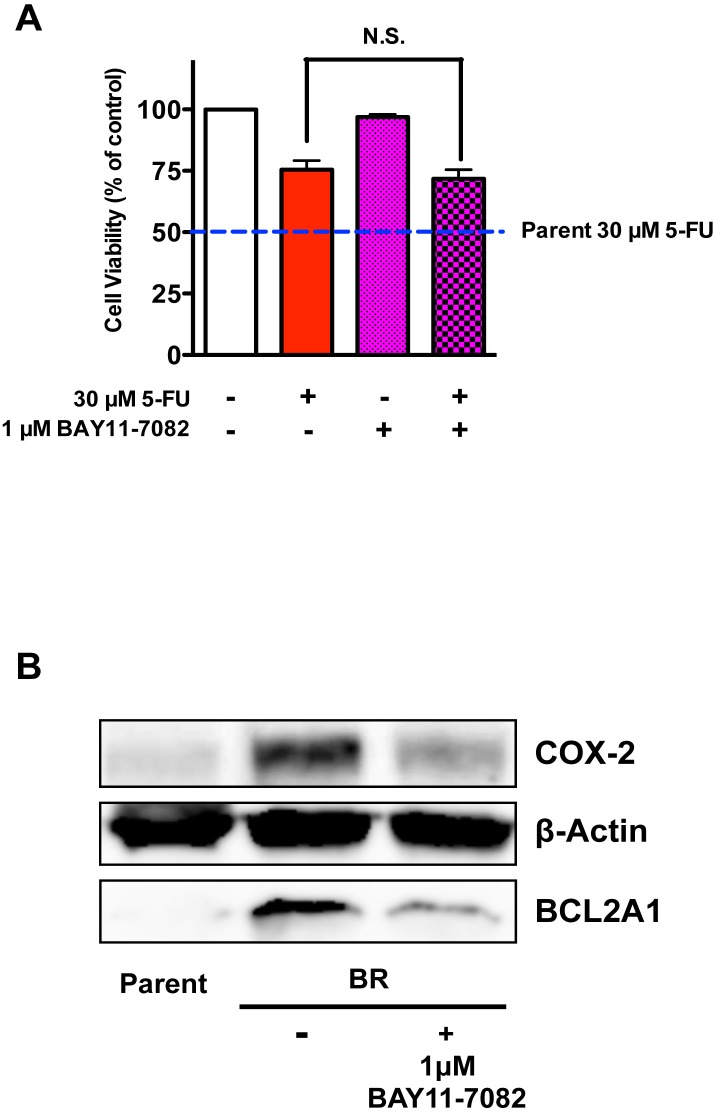
Effect of BAY11-7082 on MDA-MB-231BR as an NF-κB inhibitor. (A) Cytotoxicity of 5-FU to MDA-MB-231BR in the presence of 1 μM BAY11-7082. Blue broken lines parallel to x-axis represent the cell viability of MDA-MB-231 (Parent) treated with 30 μM 5-FU. Data represent the mean with SEM of four independent samples. (B) Changes in protein expressions of COX-2 and BCL2A1 in MDA-MB-231BR in response to 1 μM BAY11-7082.

To further investigate the extent to which elevated COX-2 and BCL2A1 expressions may have contributed to 5-FU-resistance in MDA-MB-231BR, we sought to restore the sensitivity of MDA-MB-231BR to 5-FU using different types of modulators. Celecoxib, a selective inhibitor of COX-2, could not sensitize MDA-MB-231BR to 5-FU (Fig 4). We also confirmed that celecoxib did not affect the expressions of COX-2/BCL2A1 mRNA and protein in MDA-MB-231BR (S3 Fig). Similarly, 1 μM BAY11-7082, an NF-κB inhibitor, failed to sensitize MDA-MB-231BR to 5-FU (Fig 5A), although 1 μM BAY11-7082 significantly suppressed COX-2 expression in MDA-MB-231BR up to the level in MDA-MB-231, as well as BCL2A1 expression, which was still 150-fold higher than that in MDA-MB-231 (Fig 5B and S4 Fig).

**Fig 6:**
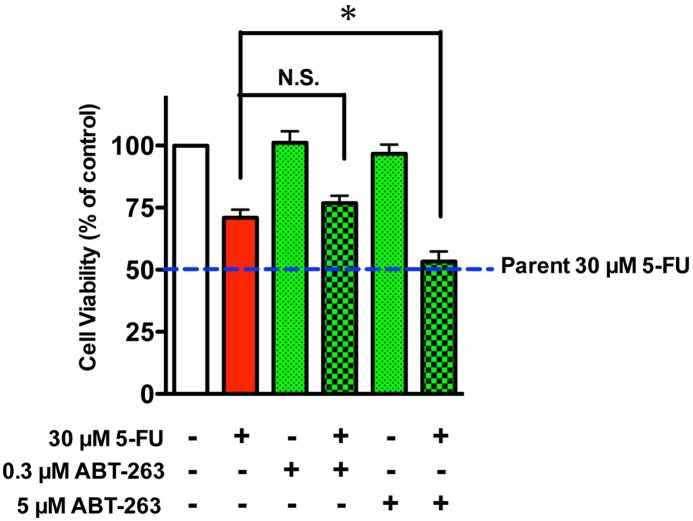
Effect of ABT-263 on the cytotoxicity of 5-FU against MDA-MB-231BR. Cytotoxicity of 5-FU to MDA-MB-231BR in the presence of 0.3 or 5 μM ABT-263. Blue broken lines parallel to x-axis represent the cell viability of MDA-MB-231 (Parent) treated with 30 μM 5-FU. Data represent the mean with SEM of four independent samples (*P<0.05, BR treated with 30 μM 5-FU *vs*. BR treated with 30 μM 5-FU and 5 μM ABT-263).

**Fig 7:**
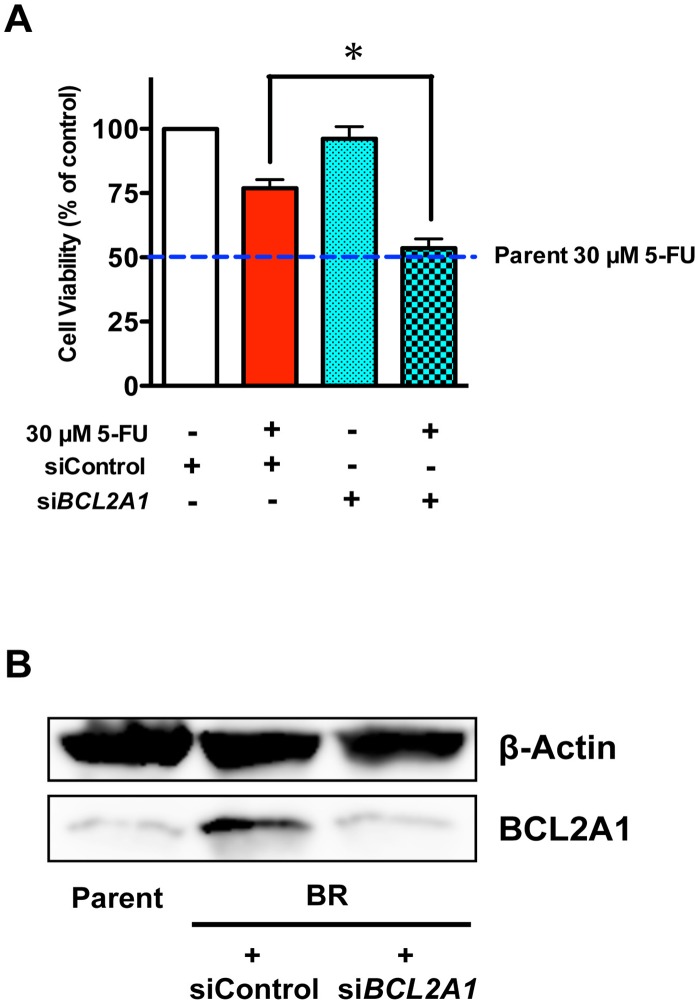
Effect of specific knockdown of *BCL2A1* on MDA-MB-231BR. (A) Cytotoxicity of 5-FU to MDA-MB-231BR after transient transfection with *BCL2A1* siRNA. Blue broken lines parallel to x-axis represent the cell viability of MDA-MB-231 (Parent) treated with 30 μM 5-FU. Data represent the mean with SEM of three independent samples (*P<0.05, BR treated with 30 μM 5-FU and siControl *vs*. BR treated with 30 μM 5-FU and si*BCL2A1*). (B) Changes in protein expression of BCL2A1 in MDA-MB-231BR in response to *BCL2A1* siRNA.

**Fig 8:**
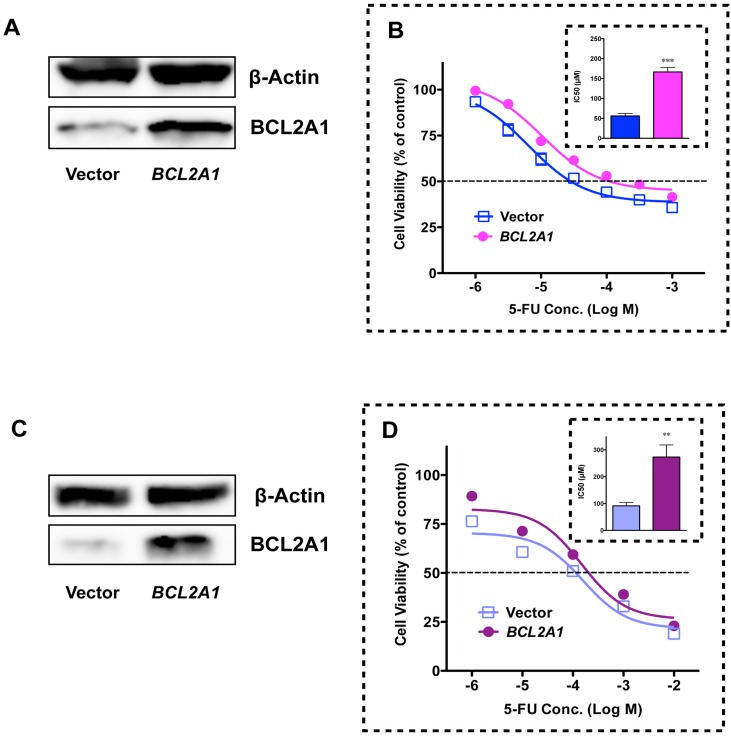
Effect of *BCL2A1* overexpression on 5-FU-resistance in MDA-MB-231 and T-47D. BCL2A1 protein expression of (A) MDA-MB-231 and (C) T-47D after transfection with a pCMV-Myc-DDK Entry vector (Vector) or a pCMV-Myc-DDK *BCL2A1* vector (*BCL2A1*). Cytotoxicity of 5-FU toward (B) MDA-MB-231 transfected with a *BCL2A1* vector and (D) T-47D transfected with a *BCL2A1* vector. The insets show the IC_50_ of 5-FU against cell lines transfected with vectors. Data represent the mean with SEM of at least three independent samples (***p<0.001 *vs*. MDA-MB-231 transfected with a control vector; *p<0.05 *vs*. T-47D transfected with a control vector).

To verify the role that BCL2A1 plays in 5-FU-resistance in MDA-MB-231BR, we used the pan-BCL2-family inhibitor ABT-263. ABT-263 binds with very high affinity to BCL2, BCL2-xL, and BCL2-w (>1 nM), but with much lower affinity to Mcl-1 and BCL2A1 (>354 nM) [6, 7]. ABT-263 did not increase the sensitivity of MDA-MB-231BR to 5-FU at a low dose (0.3 μM), but did sensitize MDA-MB-231BR to 5-FU to a level comparable to that in MDA-MB-231 at a dose of 5 μM where ABT-263 alone did not affect cell viability (Fig 6). As expected based on the inhibitory mechanism of ABT-263, i.e., disruption of intracellular BCL2 family protein-protein interactions, ABT-263 did not affect either COX-2/BCL2A1 mRNA or protein expressions in MDA-MB-231BR (S5 Fig). *BCL2A1* siRNA also sensitized MDA-MB-231BR to 5-FU to a level similar to that in MDA-MB-231 (Fig 7A), which was consistent with the phenomenal suppression of *BCL2A1* (Fig 7B and S6 Fig). Conversely, when *BCL2A1* was overexpressed in the MDA-MB-231 and T-47D cell lines, both MDA-MB-231 and T-47D cell lines developed resistance to 5-FU (Fig 8 and S7 Fig).

### BCL2A1 gene/protein expressions in MDA-MB-231BR were higher than those in other human derived cell lines

**Fig 9:**
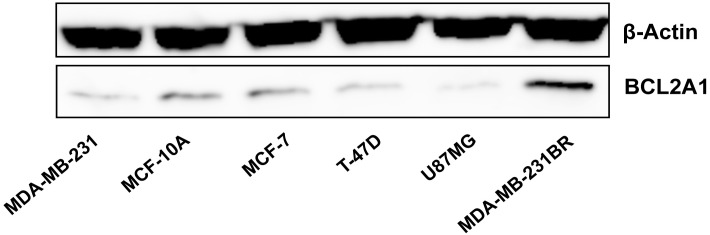
Protein expression of BCL2A1 in various human cell lines.

To explore the BCL2A1 expression level in MDA-MB-231BR relative to other cell lines, we evaluated BCL2A1 mRNA and protein expressions in other human cell lines. As predicted from an earlier report [8], both BCL2A1 gene and proteins were expressed only marginally in other human breast cancer cell lines, such as MCF-7 and T-47D, a human mammary epithelial cell line MCF-10A, and a human glioblastoma cell line U87MG (Fig 9 and S8 Fig).

## Discussion

Breast cancer supposedly has a subpopulation of refractory cancer cells [9] that slip past anticancer agents, and metastasize to other parts of the body, while vulnerable breast cancer cells metastasize to distant organs and may acquire drug resistance by genetic changes under the new microenvironment of a metastatic niche [10, 11]. In either case, metastatic breast cancer cells often show drug resistance even without prior exposure to anticancer agents. In the present study, we found that MDA-MB-231BR, a brain metastatic variant of a human breast cancer cell line, was refractory to treatment with 5-FU, while its parent cell line, MDA-MB-231, did not have the same level of resistance. Since MDA-MB-231BR and MDA-MB-231SCP2 have not been treated with any kinds of anticancer agents, the resistance of MDA-MB-231BR to 5-FU is intrinsic, and entirely distinct from conventional drug resistance. Interestingly, the sensitivity of the bone metastatic variant MDA-MB-231SCP2 to 5-FU was comparable to that of MDA-MB-231. Taken together, these metastatic phenotype-specific differences in 5-FU-resistance may be a key feature of the brain tropism of breast cancer metastasis. Alternatively, it is also conceivable that primary breast cancer cells that metastasize to the brain are genetically changed by the brain's unique microenvironment [10, 12].

We next performed RNA sequencing using these cell lines to investigate the mechanism underlying 5-FU-resistance in MDA-MB-231BR. The outcome of RNA sequencing indicated little difference in the expression of genes related to 5-FU metabolism and drug efflux transporters, both of which are commonly considered to be the main contributors to drug resistance. It has also been reported that COX-2 may be involved in 5-FU-resistance [13, 14] and brain metastasis of breast cancer [15]. In the present study, we found the increased expression of *COX-2* in MDA-MB-231BR. However, a selective COX-2 inhibitor celecoxib did not sensitize MDA-MB-231BR to 5-FU. Furthermore, the NF-κB inhibitor BAY11-7082 failed to sensitize MDA-MB-231BR to 5-FU despite the reduced expression of *COX-2*. These findings indicate that *COX-2* might not be a determining factor in 5-FU-resistance in MDA-MB-231BR.

A member of the BCL2 family, BCL2A1 is known to contribute to drug resistance [8, 16–18] through a reduction in the release of pro-apoptotic cytochrome c from mitochondria and the inhibition of caspase activation [18]. In this study, we found that the mRNA/protein expressions of BCL2A1 was dramatically elevated in MDA-MB-231BR. With regard to 5-FU-resistance, the pan-BCL2-family inhibitor ABT-263 did not sensitize MDA-MB-231BR to 5-FU at a dose at which ABT-263 could bind to BCL2, BCL2-xL, and BCL2-w, but not to BCL2A1. In stark contrast, high-dose treatment (5 μM of ABT-263), which could inhibit all BCL2-family including BCL2A1, sensitized MDA-MB-231BR to 5-FU to a level comparable to that in MDA-MB-231. More interestingly, we also found that MDA-MB-231BR indeed demonstrated resistance to ABT-263 in addition to 5-FU on the grounds that ABT-263 alone affected the viability of MDA-MB-231 at a concentration of 5 μM where the viability of MDA-MB-231BR was not affected by ABT-263 alone (S9 Fig). Furthermore, knockdown of *BCL2A1* gene expression sensitized MDA-MB-231BR to 5-FU, whereas overexpression of the *BCL2A1* gene in MDA-MB-231 by transient transfection conferred 5-FU-resistance on MDA-MB-231. These findings strongly indicate that *BCL2A1* plays a key role in the intrinsic resistance of MDA-MB-231BR to 5-FU.

**Fig 10:**
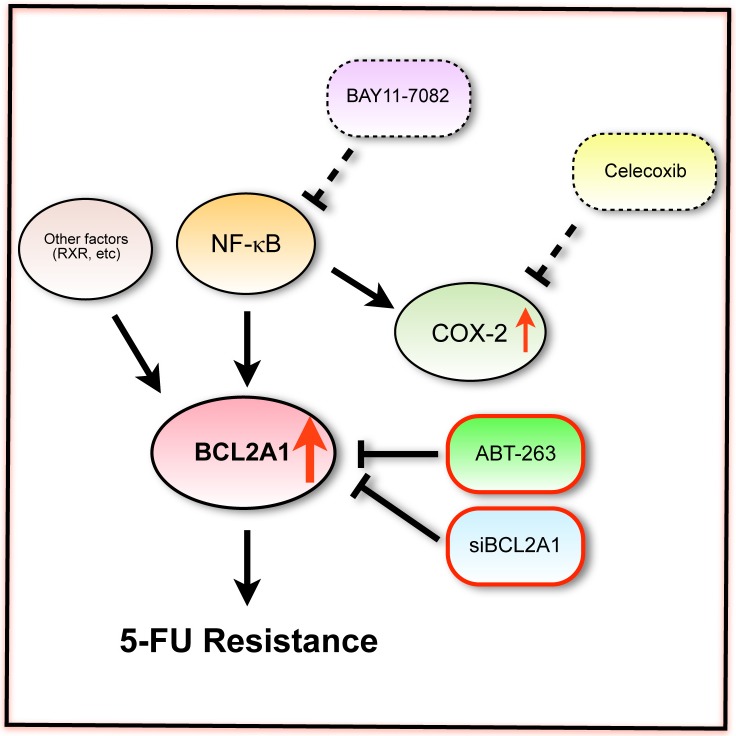
Schematic of the relationship between *BCL2A1*, its upstream regulators, and modifiers used in this study. Celecoxib acts on COX-2 but did not regulate *BCL2A1* expression, while BAY11-7082 acts directly on NF-κB and moderately regulated *BCL2A1* expression, and neither of these sensitized MDA-MB-231BR to 5-FU. Both ABT-263 and *BCL2A1* siRNA directly regulate BCL2A1, and were effective for the sensitization of MDA-MB-231BR to 5-FU.

It has been widely accepted that NF-κB is an important transcription factor of the apoptosis regulator BCL2 family [17] (Fig 10), which was the rationale behind the use of an NF-κB inhibitor BAY11-7082 in this study to inhibit the expressions of BCL2A1 mRNA/protein. The gene expression of *BCL2A1* in MDA-MB-231BR was significantly decreased by BAY11-7082, but the *BCL2A1* level was still significantly higher than that in its parent cell line. This resulted in marginal sensitization of MDA-MB-231BR to 5-FU, which was opposite to complete sensitization by *BCL2A1* siRNA that suppressed BCL2A1 mRNA/protein up to a level comparable to those in MDA-MB-231. The present fact indicates that other upstream signaling molecules of BCL2A1 transcription, such as retinoid X receptors (RXR) and Wilms' tumor 1 suppressor gene (WT1) [17], may promote the upregulation of BCL2A1 in MDA-MB-231BR. Our results along with those from earlier studies strongly support the idea that BCL2A1 plays a crucial role in 5-FU-resistance in MDA-MB-231BR.

An earlier report demonstrated that *BCL2A1* was more highly expressed in advanced breast cancers compared to less-advanced cancers in clinical situations, implying that *BCL2A1* expression could be a poor prognostic factor [19]. Since brain metastasis typically occurs in the late stage of breast cancers, elevated BCL2A1 expression in MDA-MB-231BR is a reasonable observation, although the details need to be elucidated. An *in vivo* study on 5-FU-resistance using mice bearing MDA-MB-231BR or MDA-MB-231 xenograft is underway to support our *in vitro* observation.

To the best of our knowledge, MDA-MB-231 is one of very few human breast cancer cell lines that have established metastatic variants with organ tropism, such as bone- and brain-seeking phenotypes. MDA-MB-231BR is the most often used variant that is characterized by brain metastatic potential, and has even been used in mouse models of breast cancer brain metastasis. Therefore, the outcome demonstrated in this report is not only new and significant, but should also promote further elucidation of the correlation between breast cancer metastasis and drug resistance.

In summary, we have demonstrated, for the first time, that the drug sensitivity of MDA-MB-231BR was distinct from that of MDA-MB-231SCP2 despite the same origin (MDA-MB-231). The pan-BCL2 family inhibitor ABT-263 and *BCL2A1* siRNA regulated BCL2A1 proteins, which restored the sensitivity of MDA-MB-231BR to 5-FU. Currently, a few BCL2-family inhibitors, including ABT-263 (Navitoclax), are under evaluation in clinical trials for various types of cancers [20, 21]. This could offer hope to breast cancer patients with brain metastasis who have few treatment options. Although the extent to which our observations correlate with the clinical situation is still undetermined, and these findings regarding the intrinsic resistance of MDA-MB-231BR mark the significant first step toward a better understanding of drug resistance of metastatic breast cancers. Further investigations are needed to decipher the emergence of drug resistance and organ tropism in breast cancer metastasis.

## Supporting Information

S1 Fig Cytotoxicity of doxorubicin against MDA-MB-231 and its metastatic variants. The inset shows the IC_50_ of doxorubicin against each cell line. Data represent the mean with SEM of five independent experiments. No significant differences were detected.
(EPS)

S2 Fig BCL2A1 protein expression in MDA-MB-231 and its metastatic variants quantitated by ELISA. Data represent the mean with SEM of at least five independent samples (*p<0.05 *vs*. Parent).
(EPS)

S3 Fig Effect of celecoxib on gene and protein expressions of COX-2 and BCL2A1 in MDA-MB-231BR. Changes in gene expressions of (A) *COX-2* and (B) *BCL2A1* in MDA-MB-231BR in response to 50 μM celecoxib. Gene expression levels were expressed relative to the value in Parent calculated by the 2^-ΔΔCT^ method. Data represent the mean with SEM of three independent samples. (C) Changes in protein expressions of COX-2 and BCL2A1 in MDA-MB-231BR in response to 50 μM celecoxib.
(EPS)

S4 Fig Effect of BAY11-7082 on gene expressions of *COX-2* and *BCL2A1* in MDA-MB-231BR. Changes in gene expressions of (A) *COX-2* and (B) *BCL2A1* in MDA-MB-231BR in response to 1 μM BAY11-7082. Gene expression levels were expressed relative to the value in Parent calculated by the 2^-ΔΔCT^ method. Data represent the mean with SEM of three independent samples (**P<0.01 and ***P<0.001, BR without BAY11-7082 *vs*. BR treated with BAY11-7082; ^##^*P*<0.01, Parent *vs*. BR treated with BAY11-7082).
(EPS)

S5 Fig Effect of ABT-263 on gene and protein expressions of COX-2 and BCL2A1 in MDA-MB-231BR. Changes in gene expressions of (A) *COX-2* and (B) *BCL2A1* in MDA-MB-231BR in response to 5 μM ABT-263. Gene expression levels were expressed relative to the value in Parent calculated by the 2^-ΔΔCT^ method. Data represent the mean with SEM of three independent samples. (C) Changes in protein expressions of COX-2 and BCL2A1 in MDA-MB-231BR in response to 5 μM ABT-263.
(EPS)

S6 Fig Effect of *BCL2A1* siRNA on gene expression of *BCL2A1* in MDA-MB-231BR. Gene expression levels were expressed relative to the value in Parent calculated by the 2^-ΔΔCT^ method. Data represent the mean with SEM of three independent samples (***P<0.001, BR treated with siControl *vs*. BR treated with si*BCL2A1*).
(EPS)

S7 Fig Gene expression of *BCL2A1* in MDA-MB-231 and T-47D after transfection with a *BCL2A1* vector. *BCL2A1* gene expression of (A) MDA-MB-231 and (B) T-47D after transfection with a pCMV-Myc-DDK Entry vector (Vector) or a pCMV-Myc-DDK *BCL2A1* vector (*BCL2A1*). Data represent the mean with SEM of at least three independent samples (***p<0.001 *vs*. MDA-MB-231 or T-47D transfected with a control vector).
(EPS)

S8 Fig Gene expression of *BCL2A1* in various human cell lines. Data represent the mean with SEM of at least three independent samples (***p<0.001 *vs*. MDA-MB-231BR). Relative to MDA-MB-231BR, the cell lines used in this study show negligible gene expression of *BCL2A1*.
(EPS)

S9 Fig Cytotoxic effect of ABT-263 against MDA-MB-231. Data represent the mean with SEM of three independent experiments. While ABT-263 did not affect the viability of MDA-MB-231BR even at a concentration of 5 μM (Fig 6), almost 80% of MDA-MB-231 cells were killed by ABT-263 alone at the same concentration. This indicates that MDA-MB-231BR is not only refractory to 5-FU treatment, but also to pan-BCL2-family inhibitor ABT-263 treatment.
(EPS)

S1 Table PCR primers used in this study. (DOCX)

## Abbreviations

BCL2: B-cell lymphoma 2
COX-2: cyclooxygenase-2
5-FU: 5-fluorouracil
PCR: polymerase chain reaction
TNBC: triple-negative breast cancer

## References

[1] Siegel RL, Miller KD, Jemal A. Cancer statistics, 2016. CA Cancer J Clin. 2016; 66: 7–30. 10.3322/caac.21332 26742998

[2] Wang Y, Zhou BP. Epithelial-mesenchymal transition in breast cancer progression and metastasis. Chin J Cancer. 2011; 30: 603–611. 10.5732/cjc.011.10226 21880181 PMC3702729

[3] Yamauchi K, Yang M, Hayashi K, Jiang P, Yamamoto N, Tsuchiya H, et al Induction of cancer metastasis by cyclophosphamide pretreatment of host mice: an opposite effect of chemotherapy. Cancer Res. 2008; 68: 516–520. 10.1158/0008-5472.can-07-3063 18199547

[4] Lee MS, Kim HP, Kim TY, Lee JW. Gefitinib resistance of cancer cells correlated with TM4SF5-mediated epithelial-mesenchymal transition. Biochim Biophys Acta. 2012; 1823: 514–523. 10.1016/j.bbamcr.2011.11.017 22178131

[5] Gopalan A, Yu W, Sanders BG, Kline K. Eliminating drug resistant breast cancer stem-like cells with combination of simvastatin and gamma-tocotrienol. Cancer Lett. 2013; 328: 285–296. 10.1016/j.canlet.2012.10.003 23063651

[6] Vogler M, Dinsdale D, Dyer MJ, Cohen GM. Bcl-2 inhibitors: small molecules with a big impact on cancer therapy. Cell Death Differ. 2009; 16: 360–367. 10.1038/cdd.2008.137 18806758

[7] Tse C, Shoemaker AR, Adickes J, Anderson MG, Chen J, Jin S, et al ABT-263: a potent and orally bioavailable Bcl-2 family inhibitor. Cancer Res. 2008; 68: 3421–3428. 10.1158/0008-5472.CAN-07-5836 18451170

[8] Haq R, Yokoyama S, Hawryluk EB, Jonsson GB, Frederick DT, McHenry K, et al BCL2A1 is a lineage-specific antiapoptotic melanoma oncogene that confers resistance to BRAF inhibition. Proc Natl Acad Sci U S A. 2013; 110: 4321–4326. 10.1073/pnas.1205575110 23447565 PMC3600451

[9] Hiraga T, Ito S, Nakamura H. Side population in MDA-MB-231 human breast cancer cells exhibits cancer stem cell-like properties without higher bone-metastatic potential. Oncol Rep. 2011; 25: 289–296. 10.3892/or_00001073 21109989

[10] Kim SJ, Kim JS, Park ES, Lee JS, Lin Q, Langley RR, et al Astrocytes upregulate survival genes in tumor cells and induce protection from chemotherapy. Neoplasia. 2011; 13: 286–298. 10.1593/neo.11112 21390191 PMC3050871

[11] Sierra A, Price JE, Garcia-Ramirez M, Mendez O, Lopez L, Fabra A. Astrocyte-derived cytokines contribute to the metastatic brain specificity of breast cancer cells. Lab Invest. 1997; 77: 357–368. 9354770

[12] Chen Q, Boire A, Jin X, Valiente M, Er EE, Lopez-Soto A, et al Carcinoma-astrocyte gap junctions promote brain metastasis by cGAMP transfer. Nature. 2016; 533: 493–498. 10.1038/nature18268 27225120 PMC5021195

[13] Okamura H, Fujiwara H, Umehara S, Okamura S, Todo M, Furutani A, et al COX-2 overexpression induced by gene transfer reduces sensitivity of TE13 esophageal carcinoma cells to 5-fluorouracil and cisplatin. Anticancer Res. 2013; 33: 537–542. 23393346

[14] Hwang JT, Ha J, Park OJ. Combination of 5-fluorouracil and genistein induces apoptosis synergistically in chemo-resistant cancer cells through the modulation of AMPK and COX-2 signaling pathways. Biochem Biophys Res Commun. 2005; 332: 433–440. 10.1016/j.bbrc.2005.04.143 15896711

[15] Bos PD, Zhang XH, Nadal C, Shu W, Gomis RR, Nguyen DX, et al Genes that mediate breast cancer metastasis to the brain. Nature. 2009; 459: 1005–1009. 10.1038/nature08021 19421193 PMC2698953

[16] Cheng Q, Lee HH, Li Y, Parks TP, Cheng G. Upregulation of Bcl-x and Bfl-1 as a potential mechanism of chemoresistance, which can be overcome by NF-κB inhibition. Oncogene. 2000; 19: 4936–4940. 10.1038/sj.onc.1203861 11039911

[17] Vogler M. BCL2A1: the underdog in the BCL2 family. Cell Death Differ. 2012; 19: 67–74. 10.1038/cdd.2011.158 22075983 PMC3252829

[18] Wang CY, Guttridge DC, Mayo MW, Baldwin AS Jr. NF-κB induces expression of the Bcl-2 homologue A1/Bfl-1 to preferentially suppress chemotherapy-induced apoptosis. Mol Cell Biol. 1999; 19: 5923–5929. 10454539 10.1128/mcb.19.9.5923 PMC84448

[19] Yoon HS, Hong SH, Kang HJ, Ko BK, Ahn SH, Huh JR. Bfl-1 gene expression in breast cancer: its relationship with other prognostic factors. J Korean Med Sci. 2003; 18: 225–230. 10.3346/jkms.2003.18.2.225 12692420 PMC3055024

[20] Gandhi L, Camidge DR, Ribeiro de Oliveira M, Bonomi P, Gandara D, Khaira D, et al Phase I study of Navitoclax (ABT-263), a novel Bcl-2 family inhibitor, in patients with small-cell lung cancer and other solid tumors. J Clin Oncol. 2011; 29: 909–916. 10.1200/JCO.2010.31.6208 21282543 PMC4668282

[21] Roberts AW, Seymour JF, Brown JR, Wierda WG, Kipps TJ, Khaw SL, et al Substantial susceptibility of chronic lymphocytic leukemia to BCL2 inhibition: results of a Phase I study of navitoclax in patients with relapsed or refractory disease. J Clin Oncol. 2012; 30: 488–496. 10.1200/JCO.2011.34.7898 22184378 PMC4979082

