# Supplementary material for: Intrinsic Resistance to 5-Fluorouracil in a Brain Metastatic Variant of Human Breast Cancer Cell Line, MDA-MB-231BR
Source: PLoS One. 2016 Oct 10;11(10):e0164250. doi: 10.1371/journal.pone.0164250 (PMC5056764; doi:10.1371/journal.pone.0164250)
Supplement: S1 Table — (DOCX) [file pone.0164250.s010.docx]

**S1 Table. PCR primers used in this study.**

|  | Accession number |  | Primers sequences | Product size (base pairs) |
| --- | --- | --- | --- | --- |
| RPS18 | NM_022551.2 | Forward | ATACAGCCAGGTCCTAGCCA | 96 |
|  |  | Reverse | AAGTGACGCAGCCCTCTATG |  |
| COX-2 | NM_000963.3 | Forward | GCTACAAAAGCTGGGAAGCC | 109 |
|  |  | Reverse | AGCTGCTTTTTACCTTTGACACC |  |
| BCL2A1 | NM_004049.3 | Forward | AAATTGCCCCGGATGTGGAT | 115 |
|  |  | Reverse | ACAAAGCCATTTTCCCAGCC |  |
